# Artificial intelligence in ADHD: a global perspective on research hotspots, trends and clinical applications

**DOI:** 10.3389/fnhum.2025.1577585

**Published:** 2025-04-10

**Authors:** Xiaofang Wang, Qianfang Jia, Lvyuan Liang, Weiwei Zhou, Weihua Yang, Jingfeng Mu

**Affiliations:** ^1^Hebei University of Chinese Medicine, Shijiazhuang, China; ^2^Department of Children's Rehabilitation, First Affiliated Hospital of Xinxiang Medical University, Xinxiang, China; ^3^Xinxiang Autism Integration Education Engineering and Technology Research Center, Xinxiang, China; ^4^School of Pediatric Medicine, Henan University of Chinese Medicine, Zhengzhou, China; ^5^Shenzhen Eye Hospital, Shenzhen Eye Medical Center, Southern Medical University, Shenzhen, China

**Keywords:** artificial intelligence, attention deficit hyperactivity disorder, diagnosis, hotspot, trend

## Abstract

**Background:**

Artificial Intelligence (AI), has garnered attention in research on attention deficit hyperactivity disorder (ADHD). In the future, AI may have clinical applications in ADHD, particularly in facilitating the objective diagnosis and classification of ADHD. This study aimed to comprehensively analyze the current status and research frontiers of AI applications in ADHD, identifying hotspots and trends to guide future research directions and promote clinical advancements in this field.

**Methods:**

Articles in the field of AI applications in ADHD were from the Web of Science Core Collection (WoSCC) database. Analysis was conducted using CiteSpace 6.3.R.1. Additionally, high-impact articles were analyzed.

**Results:**

A total of 342 articles from 50 countries and regions were included. The United States led with 103 articles, having the highest H-index of 21, followed by China with 69 articles, and England with 34 articles. The State University of New York System produced the most articles (11), and *Frontiers in Psychiatry* had the most articles (12). Burst keywords in 2022–2024 included “diagnosis,” “network,” “attention deficit hyperactivity disorder” and “artificial intelligence.”

**Conclusion:**

AI technologies have become a prominent topic in ADHD research, with the United States, China, and England leading in articles and influence. The State University of New York System was the most influential institution, while *Frontiers in Psychiatry* stood out as the key journal. Utilizing networks and other AI technologies for diagnosing ADHD represents current hotspots and future trends, potentially offering objective indicators for ADHD.

## 1 Introduction

Artificial Intelligence (AI) has been widely employed in the medical field for research and clinical practice (Gong et al., 2024; Yan et al., 2024; Zhang et al., 2025). Notably, it plays a significant role in neurology and psychiatry (Pham et al., 2022; McCutcheon et al., 2025). In these disciplines, AI contributes to early detection, accurate diagnosis, and effective treatment strategies (Haug and Drazen, 2023; Jia et al., 2023; Wan et al., 2023). Among various neurodevelopmental disorders, attention deficit hyperactivity disorder (ADHD) often persists into adulthood. More recently, advanced imaging and electrophysiological techniques, such as magnetic resonance imaging (MRI) and electroencephalography (EEG), have been introduced as diagnostic indicators (Choi et al., 2023; Lohani and Rana, 2023; Eng et al., 2025). However, these methods remain susceptible to human bias (Sibley, 2021; Mulraney et al., 2022). The application of AI techniques is transforming this predicament. Advanced AI methods, such as deep learning and machine learning algorithms, can process large datasets to identify patterns and make predictions (Loh et al., 2022; Chen et al., 2023). These techniques are being applied to the screening and diagnosis of ADHD, presenting the potential to create more objective and accurate diagnostic and classification models for ADHD.

Overview of the current research, it becomes evident that the application of AI in ADHD research constitutes an important and captivating field. A comprehensive analysis can analyze articles within a specific field to provide current status, research hotspots, and future trends. By counting and analyzing citations, publications, and other metrics, it can provide insights into the development and trends of a field. Although AI and ADHD have been analyzed separately in the past using methods analogous to comprehensive analysis (Zhao et al., 2023; Liu et al., 2024), it is still essential to conduct an integrated analysis of research in both fields. This would help capture the current state of research and identify hotspots, and reveal trends in the application of AI to ADHD. Additionally, it can highlight common challenges, such as data heterogeneity, model generalizability, and clinical applicability. These findings would provide valuable insights for future research and clinical applications. In this study, we have conducted a comprehensive analysis based on articles from the Web of Science Core Collection (WoSCC) database, providing a comprehensive perspective on AI’s applications in ADHD research. Additionally, high-impact articles were evaluated individually to highlight significant findings. This study aimed to conduct a comprehensive assessment of the application of AI in ADHD research. In this study, articles from the WoSCC database were analyzed to assess the research status, hotspots, and trends in this field of AI in ADHD. Particular emphasis was placed on examining the current state and limitations of AI in ADHD diagnosis. This analysis could provide a critical foundation for future research and offer valuable guidance for medical experts, imaging specialists, and engineers in advancing the field.

## 2 Materials and methods

**Figure 1:**
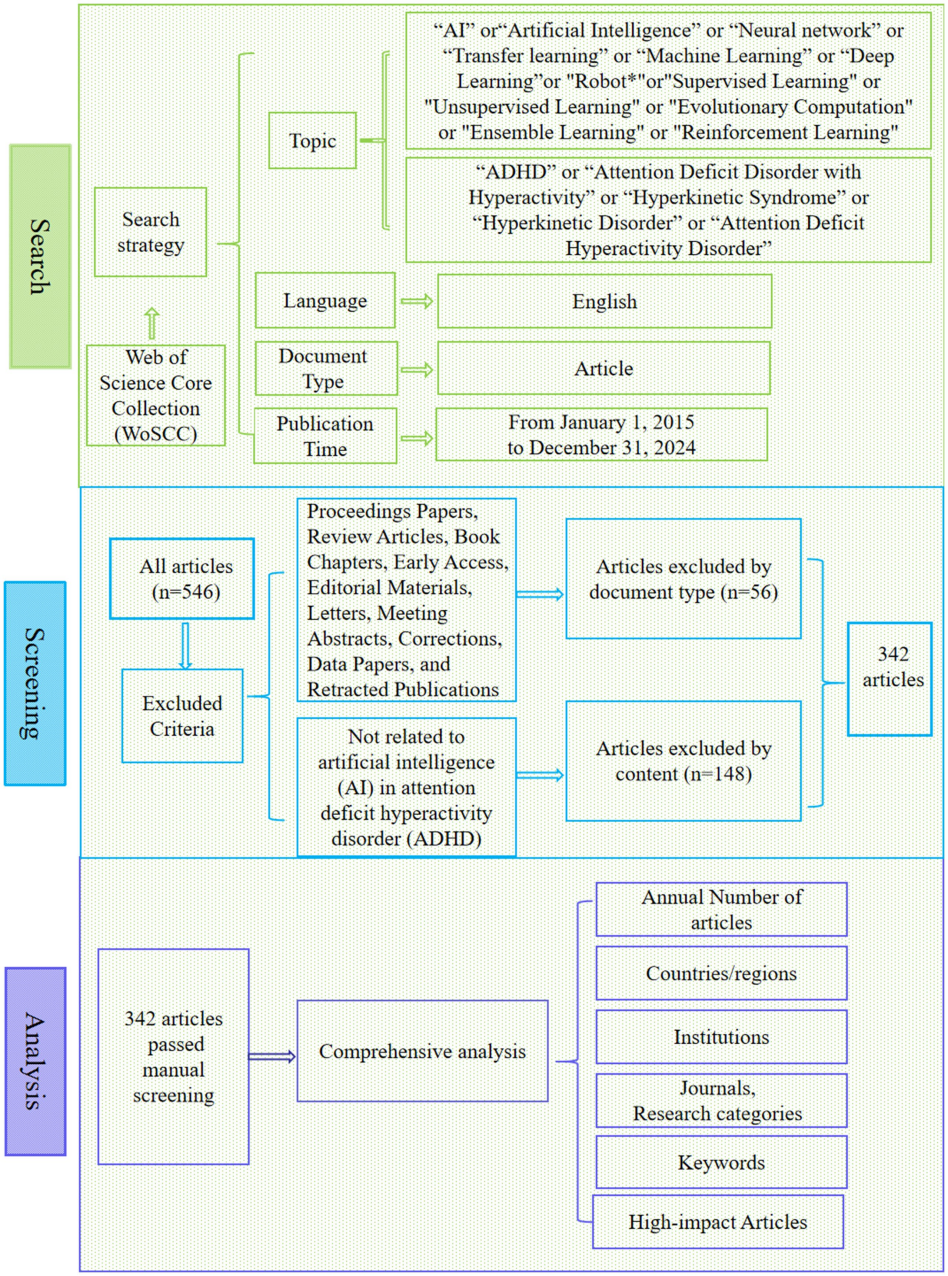
Frame flow diagram for the research on 10-year articles.

Articles in the field of AI in ADHD were searched and downloaded from the WoSCC database. The search formula was TS = (“AI” or “Artificial Intelligence” or “Neural Network” or “Transfer Learning” or “Machine Learning” or “Deep Learning” or “Robot*” or “Supervised Learning” or “Unsupervised Learning” or “Evolutionary Computation” or “Ensemble Learning” or “Reinforcement Learning” or “Large Language Model” or “LLM”) AND (“ADHD” or “Attention Deficit Disorder with Hyperactivity” or “Hyperkinetic Syndrome” or “Hyperkinetic Disorder” or “Attention Deficit Hyperactivity Disorder”). Besides, the language was limited to English, the document type was article, and the publication time was from January 1, 2015, to December 31, 2024. A total of 546 articles were retrieved and then proceeded to the manual screening stage, which included screening of both document type and content. Specific screening principles are shown in Figure 1. According to document type, articles such as proceedings papers, review articles, book chapters, and editorial materials were excluded. Subsequently, a manual screening was conducted based on the content of the articles. “Relevance” was determined based on whether the article focused on the application of AI in ADHD and its alignment with the central theme of this study. This assessment included evaluating whether the methods and techniques involved AI and whether the research subjects included individuals diagnosed with ADHD, with articles of low content relevance being eliminated. All processes were conducted by two researchers who independently determined the data (X.W. and Q.J.), and the reliability test was conducted by another researcher (W.Y.) who ruled on the article when the two former judges disagreed. CiteSpace.6.3.R.1 was employed to analyze and visualize the articles to obtain contributions and collaborative networks among countries or regions, institutions, and journals, as well as to explore trends within the data. Based on previous studies (Feng et al., 2023; Li et al., 2024, p. 2), the default parameters of the software were applied to include the most relevant and impactful articles, while enabling a detailed, year-by-year analysis of trends within the selected period. Specifically, the settings were as follows: Time slice = 2015–2024, Year Per Slice = 1, Top N% = 10.0%, and Top N = 50. Furthermore, high-impact articles were analyzed in depth. Figure 1 shows the research process in detail.

## 3 Results

### 3.1 Annual number of articles

**Figure 2:**
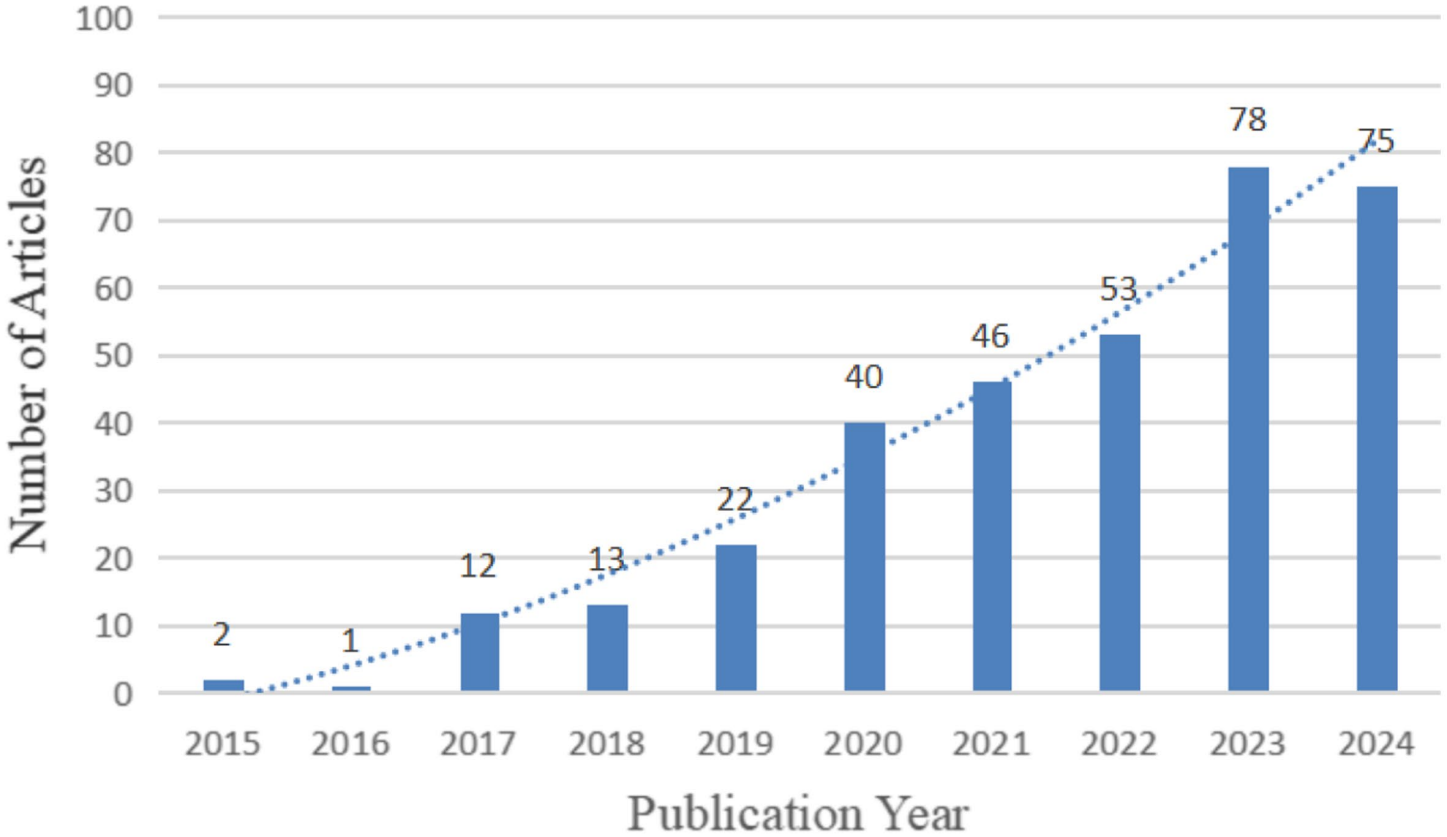
Annual number of articles in the field of AI in ADHD.

After conducting a thorough search and manual screening, a total of 342 relevant articles were identified. The dataset revealed a significant upward trend in articles since 2017, the years 2023 and 2024 marked the peak, with 78 articles in 2023 and 75 articles in 2024, respectively. This upward trend highlights the growing interest and research in the application of AI in ADHD, as depicted in Figure 2, which showcases the annual number of articles over the last decade.

### 3.2 Countries or regions

The compilation of articles spans 50 countries and regions, illustrating a wide geographical interest in this research area. According to the detailed indicators presented in Table 1, the United States held the top position in terms of article counts, centrality, and H-index, with 103 articles, a centrality of 0.38, and an H-index of 21. China followed with 69 articles and an H-index of 16. England ranked third, with 34 articles; however, it had a relatively high centrality of 0.25.

**Table 1:** Top 5 countries or regions with articles in the field of AI in ADHD.

Rank	Countries or regions	Counts	Centrality	H-index
1	United States	103	0.38	21
2	China	69	0.04	16
3	England	34	0.25	15
4	India	28	0.08	6
5	Spain	23	0.30	10

### 3.3 Institutions

**Figure 3:**
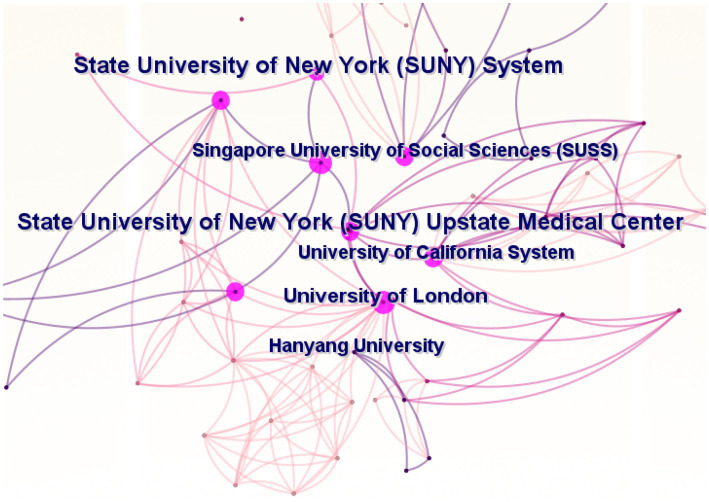
Cooperation of institutions that contributed to articles in the field of AI in ADHD.

Among institutions contributing to this field, the State University of New York System, the State University of New York Upstate Medical Center, and Harvard University, all based in the United States, were the most prolific, followed by the Singapore University of Social Sciences and the University of London, as detailed in Table 2. This table, alongside Figure 3, highlights substantial inter-institutional collaboration, demonstrating the collective effort in advancing AI research in ADHD.

**Table 2:** Top 5 Institutions with articles in the field of AI in ADHD.

Rank	Institution	Country or regions	Counts	H-index
1	State University of New York System	United States	11	8
2	State University of New York Upstate Medical Center	United States	10	7
3	Harvard University	United States	9	9
4	Singapore University of Social Sciences	Singapore	7	6
5	University of London	England	7	7

### 3.4 Journals and research categories

In terms of article outlets, *Frontiers in Psychiatry* and *IEEE Access* emerged as the foremost journals, with 12 and 11 articles published, respectively. These articles were primarily in the research categories of psychiatry, computer science, engineering, and telecommunications. Table 3 outlines these journals’ Research categories, article counts, and journal impact factor 2023.

**Table 3:** Top 5 journals of articles in the field of AI in ADHD.

Rank	Journals	Research categories	Counts	Journal impact factor 2023
1	*Frontiers in Psychiatry*	Psychiatry	12	3.2
2	*IEEE Access*	Computer Science; Engineering; Telecommunications	11	3.4
3	*Journal of Attention Disorders*	Psychology; Psychiatry	8	2.7
4	*Journal of Neural Engineering*	Engineering; Neurosciences & Neurology	8	3.7
5	*Scientific Reports*	Science & Technology	7	3.8

### 3.5 Keywords

**Figure 4:**
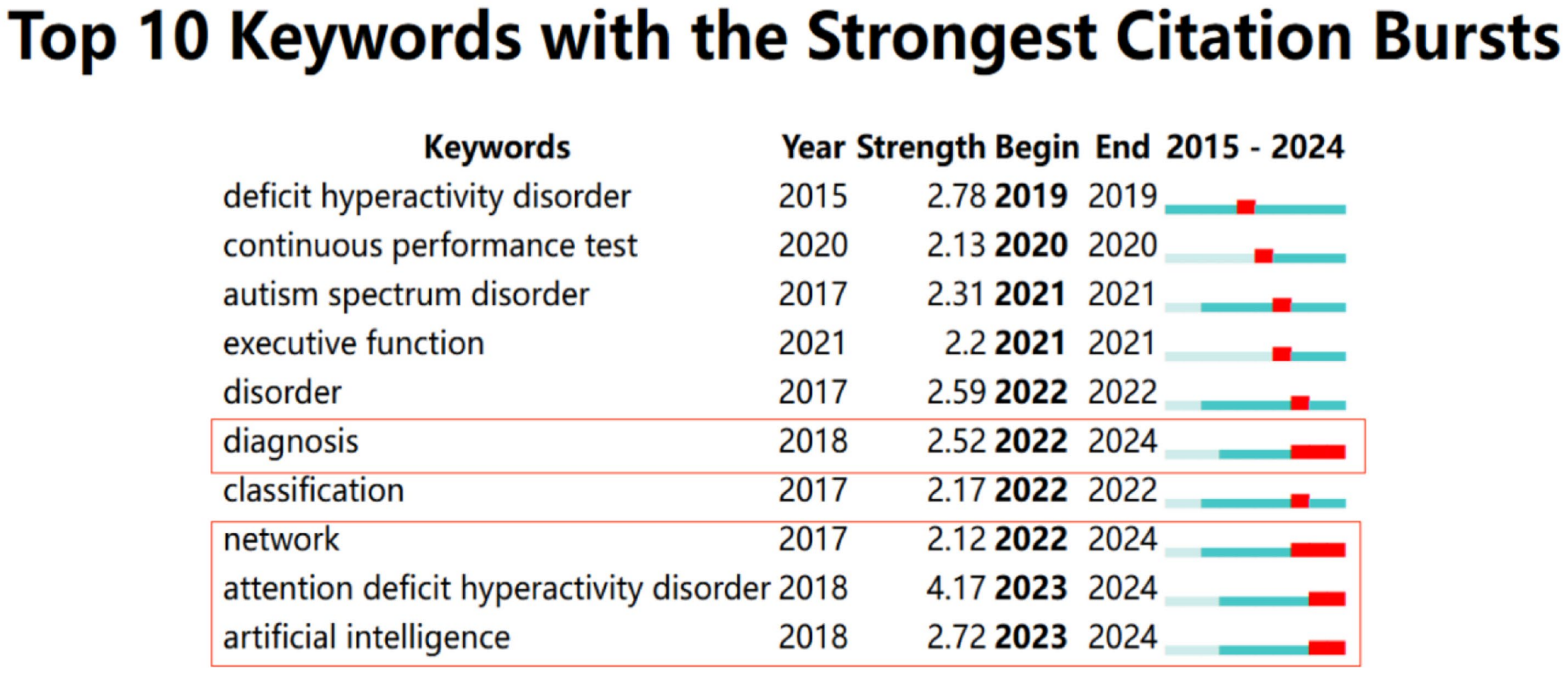
Keywords with the strongest citation bursts in the field of AI in ADHD.

**Figure 5:**
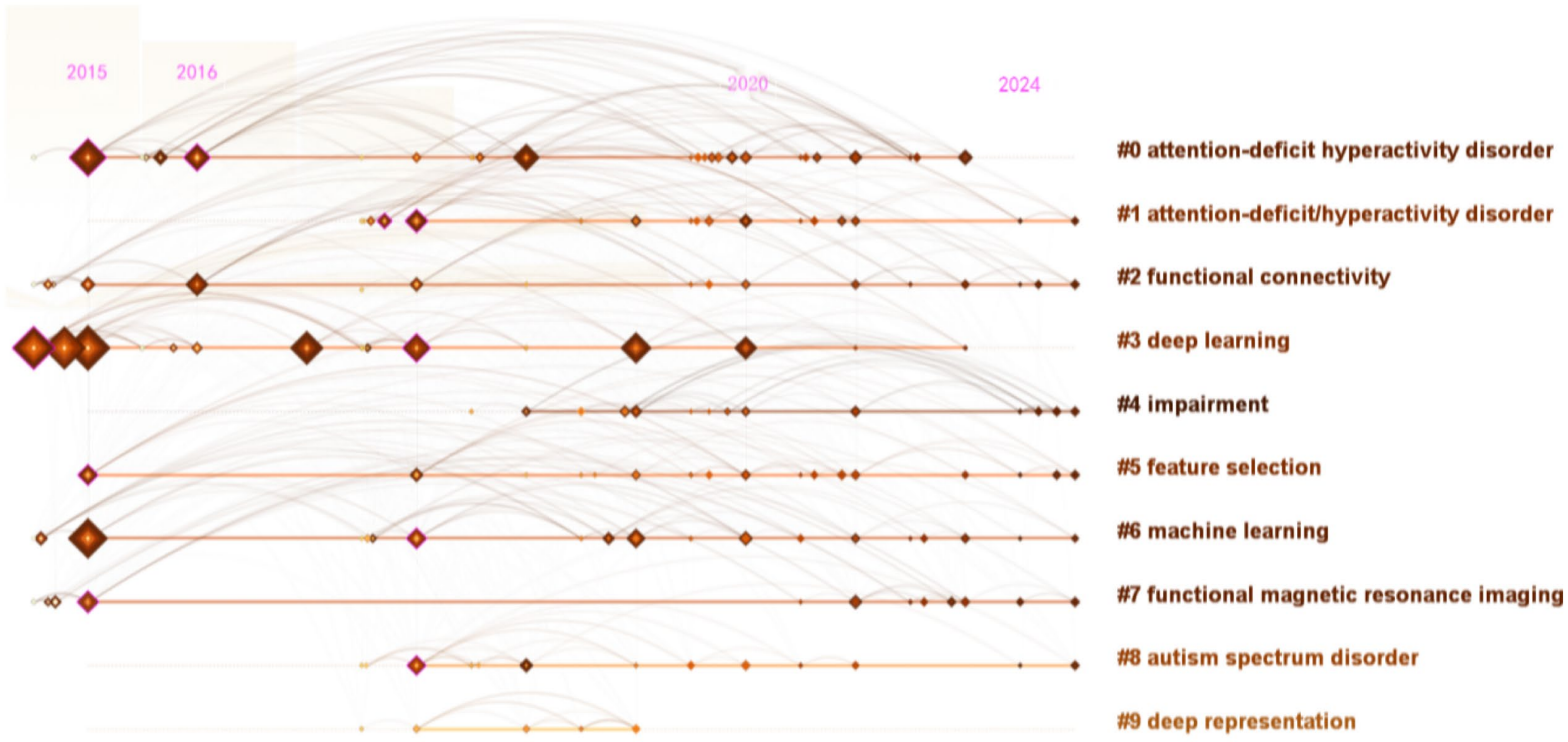
Timeline view of keywords clustering in the field of AI in ADHD.

The keywords were analyzed using CiteSpace with parameters set to “Year Per Slice” = 1, “Top N%” = 10.0%, and “Minimum Duration “= 1, and Figure 4 shows the results. The emerging burst keywords in the period of 2022–2024 were “diagnosis,” “network,” “attention deficit hyperactivity disorder” and “artificial intelligence,” which were the newest and longest-duration keywords. The default settings of CiteSpace were applied to the keywords clustering analysis, and the descending order of the cluster labels was produced in Figure 5. The top 3 clustering labels were “attention-deficit hyperactivity disorder” “attention-deficit/ hyperactivity disorder” and “functional connectivity.”

### 3.6 High-impact articles

An article with high citations is defined as a high-impact article, and Table 4 demonstrates the top 10 high-impact articles with the highest times of citations. All of these articles focused on the application of AI in the diagnosis and classification of ADHD. Among them, 5 articles were based on MRI imaging data, 3 articles utilized EEG data, and 1 article was based on behavioral assessment, with all achieving high accuracy. The AI techniques employed included machine learning, deep learning, convolutional neural network (CNN), and graph convolutional network (GCN), among others.

**Table 4:** High-impact articles in the field of AI in ADHD.

Rank	Title of article	Publication year	DOI	Times cited	Interpretation of the findings
1	3D CNN Based Automatic Diagnosis of Attention Deficit Hyperactivity Disorder Using Functional and Structural MRI	2017	10.1109/ACCESS.2017.2762703	201	The 3D CNN method was applied to MRI scans to classify ADHD, obtaining an accuracy of 69.15%.
2	Spatio-temporal deep learning method for ADHD fMRI classification	2019	10.1016/j.ins.2019.05.043	131	A deep learning method based on granular computing was proposed to analyze resting-state functional magnetic resonance imaging (rs-MRI) data to assist in the diagnosis of ADHD, and the accuracy rate reached 71.3%.
3	Use of machine learning for behavioral distinction of autism and ADHD	2016	10.1038/tp.2015.221	129	Testing of six machine learning algorithms showed that machine learning can be used for behavioral assessment to distinguish between autism and ADHD with a high degree of accuracy.
4	A deep learning framework for identifying children with ADHD using an EEG-based brain network	2019	10.1016/j.neucom.2019.04.058	110	A deep learning framework combining EEG-based brain networks and CNNs was employed to identify ADHD children with 94.67% accuracy.
5	Fully Connected Cascade Artificial Neural Network Architecture for Attention Deficit Hyperactivity Disorder Classification From Functional Magnetic Resonance Imaging Data	2015	10.1109/TCYB.2014.2379621	101	The fully connected cascade artificial neural network architecture was applied to classify ADHD from functional magnetic resonance imaging (fMRI) data with nearly 90% accuracy in diagnosing ADHD and 95% accuracy in classifying ADHD subtypes.
6	DeepFMRI: End-to-end deep learning for functional connectivity and classification of ADHD using fMRI	2020	10.1016/j.jneumeth.2019.108506	88	The end-to-end deep learning architecture was applied to classify ADHD from fMRI data with 73.1% accuracy, 91.6% specificity, and 65.5% sensitivity, highlighting the role of functional connectivity and the frontal lobe in ADHD diagnosis.
7	Fusion of fMRI and non-imaging data for ADHD classification	2018	10.1016/j.compmedimag.2017.10.002	81	Based on resting-state fMRI, a machine learning framework that combines non-imaging data with imaging data was proposed to classify ADHD with reliable classification results.
8	Diagnose ADHD disorder in children using convolutional neural network based on continuous mental task EEG	2020	10.1016/j.cmpb.2020.105738	78	The CNN-based model was applied to classify ADHD in children using EEG data based on a continuous mental task, achieving 98.48% accuracy in subject-based tests, demonstrating exceptional performance in early ADHD diagnosis.
9	Automated detection of conduct disorder and attention deficit hyperactivity disorder using decomposition and nonlinear techniques with EEG signals	2021	10.1016/j.cmpb.2021.105941	73	The automated system was performed to analyze the EEG signals to differentiate between ADHD and conduct disorder, and the results showed that the highest accuracy of 97.88% was achieved using the K-Nearest Neighbor classifier.
10	A dynamic graph convolutional neural network framework reveals new insights into connectome dysfunctions in ADHD	2022	10.1016/j.neuroimage.2021.118774	71	The dynamic graph convolutional network (dGCN) model was applied to analyze fMRI data for ADHD diagnosis, achieving 72.0% accuracy and 71.6% specificity, significantly outperforming existing methods.

## 4 Discussion

### 4.1 General data of AI in ADHD

Research into the applications of AI in ADHD has seen substantial growth, particularly after 2020, with an impressive surge to 78 articles in 2023 and 75 articles in 2024. This trend underscores AI’s increasingly central role in ADHD research. With advancements in computer technologies, methodologies such as machine learning, deep learning, and convolutional neural networks (CNNs) have become pivotal in harnessing large medical datasets for disease screening, diagnosis, and management. Significant contributions have been made in the application of AI to ADHD research. Lohani and Rana (2023) developed a machine learning framework that classifies ADHD with 75% accuracy using MRI and personal characteristics data. Khare and Acharya (2023) created AI models for ADHD detection via EEG signals, which have been noted for their high reliability and interpretability. Furthermore, Ortuno-Miro et al. (2023) applied machine learning to functional near-infrared spectroscopy (fNIRS) data for ADHD identification during mental arithmetic tasks, producing promising results. Additionally, Yoo et al. (2024) used eye-tracking techniques for diagnosis, achieving a 76.3% accuracy rate.

Based on the above data, the United States published 103 articles, with a centrality of 0.38 and an H-index of 21, establishing its dominant position in research output and global influence. China contributed 69 articles with an H-index of 16, demonstrating significant impact, although its international influence remains more localized. England published 34 articles with a centrality of 0.25, playing a crucial role in fostering interdisciplinary connections across various fields. Institutions such as the State University of New York System and Harvard University have been instrumental in advancing research. International collaborations with institutions like the Singapore University of Social Sciences and the University of London have further strengthened global research efforts. Journals such as *Frontiers in Psychiatry* and *IEEE Access* have become crucial platforms for disseminating interdisciplinary research. These journals have integrated fields of psychiatry and AI, and promoted the applications of AI in ADHD, significantly influencing both academic development and clinical practices. High-impact articles in this area mostly focused on employing AI technologies for ADHD diagnosis and classification, utilizing MRI, EEG, and other data analyzed through various AI techniques.

### 4.2 Research hotspots of AI in ADHD

“Hotspot” refers to the growing research interest in the application of AI in ADHD. Emerging keywords represent research hotspots, and the change of burst keywords over the timeline indicates the variation of hotspots, reflecting current hot topics in the field of research on AI used in ADHD.

The emerging burst keyword for 2018–2019 was “deficit hyperactivity disorder,” indicating a focus on ADHD (O’Mahony et al., 2014; Itani et al., 2019). In 2020–2020, the emerging burst keyword was “continuous performance test.” This keyword mainly referred to AI combined with continuous performance tests to assess and diagnose ADHD in terms of visual alertness and sustained attention, etc. (Kaur et al., 2020; Slobodin et al., 2020). The emerging burst keywords in 2021–2021 were “autism spectrum disorder (ASD)” and “executive function.” This trend suggested that ASD, as a co-morbidity and differential diagnosis of ADHD, have become a prominent research focus. AI techniques played a crucial role in distinguishing between the two conditions (Ardulov et al., 2021; Kushki et al., 2021). Meanwhile, the “executive function” was used as a behavioral indicator for ADHD screening and efficacy assessment (Medina et al., 2021; Oztekin et al., 2021). The emerging keywords in 2022–2023 included “disorder” and “classification.” “Disorder” referred to the applications of AI technologies in distinguishing and classifying the severity and types of disorders in ADHD patients in addition to other neurodevelopmental and mental disorders. These disorders include ASD (Kuttala et al., 2022), oppositional defiant disorder (ODD) (de Lacy and Ramshaw, 2023), conduct disorder (CD) (Koh et al., 2022), and obsessive compulsive disorder (OCD) (Kushki et al., 2019). “Classification” designated the applications of AI technologies to produce various classification models. These models were applied to MRI, EEG, electrocardiogram (ECG), and behavioral data to classify ADHD, involving classifying ADHD subtypes, ADHD and typically developing people, ADHD, and other brain disorders (Tor et al., 2021; Uyulan et al., 2023).

The emerging burst keywords for 2023–2024 were “diagnosis,” “network,” “attention deficit hyperactivity disorder” and “artificial intelligence” which represent the latest research hotspots continuing to date.

The emerging keyword “diagnosis” denoted the increasing use of AI technologies in the diagnosis of ADHD, which may change the way ADHD is diagnosed. The diagnosis of ADHD still strictly relies on clinical assessment based on behavioral symptoms such as inattention, impulsivity, and hyperactivity. This diagnosis primarily relies on diagnostic classification systems, mainly including the Diagnostic and Statistical Manual of Mental Disorders (5th edition) (DSM-5) and the International Classification of Diseases 11th edition (ICD-11). However, as the diagnostic process heavily depends on the clinician’s experience, there is a risk of misdiagnosis, underdiagnosis, or overdiagnosis, and objective diagnostic criteria are continuously being explored (Drechsler et al., 2020). MRI and EEG respond to brain structure and function through magnetic resonance techniques and electroencephalographic signals. In recent years, studies have found that there are differences in MRI, EEG, and other data in patients with ADHD in the resting state and when performing tasks (Hoogman et al., 2017; Newson and Thiagarajan, 2019; Boedhoe et al., 2020). The applications of AI technologies, such as deep learning, machine learning, and neural networks, allow the data to be analyzed to form diagnostic models to diagnose ADHD. Zhao et al. (2022) proposed a dynamic graph convolutional network for fMRI data analysis, significantly improving diagnostic performance for ADHD. Alkahtani et al. (2023) presented an EEG-based AI model to detect ADHD, achieving an accuracy of 97.75%. The utilization of AI technologies to analyze MRI, EEG and other data for accurate and objective diagnosis of ADHD is valuable for the early objective diagnosis of ADHD and may further improve the prognosis.

The emerging keyword “network” reflected the growing use of neural networks in ADHD research, including CNNs, artificial neural networks, deep neural networks, and temporal convolutional networks, to simulate human brain neural networks. This AI technique has yielded a high-accuracy classification of ADHD and its subtypes, as well as distinguished ADHD from other disorders. Neural networks, alongside machine learning methods applied to functional connectivity data such as the default mode network, have emerged as powerful tools in ADHD screening and identification. Alves et al. (2024) performed neural networks to classify ADHD and subtypes based on event-related point locations and achieved an average accuracy of 99.72 and 99.31%, respectively. Loh et al. (2023) analyzed ECG features using a convolutional network model to classify ADHD and CD with accuracy and sensitivity above 90%. Wang et al. (2023) proposed a temporal convolutional network to analyze the dynamic kinetic connection of MRI and obtained a better diagnostic performance for ADHD. In addition, “network” is also associated with “default mode network.” There are features of default mode network in functional connectivity of patients with ADHD (Kumar et al., 2021; Hwang et al., 2025), and AI techniques are utilized to extract these features and further screen for ADHD. Neural networks have become a widely used AI technique in ADHD research and may be applied to clinical diagnosis of ADHD in the future.

The emerging keywords “attention deficit hyperactivity disorder” and “artificial intelligence” are highly relevant to the theme of this study. As the standardized term for ADHD has been widely recognized, the application of AI technologies in screening, classification, and comorbidity differentiation has demonstrated increasing reliability. The disorder was first introduced in the second edition of the *Diagnostic and Statistical Manual of Mental Disorders* (DSM-II) in 1968 under the name “Hyperkinetic Reaction of Childhood.” In 1980, the third edition (DSM-III) formally redefined the condition as “Attention Deficit Disorder (ADD).” Subsequent revisions incorporated the term “Hyperactivity,” ultimately establishing the current designation “Attention Deficit Hyperactivity Disorder (ADHD) (Kelly, 2018; Pruim et al., 2019).” The fifth edition of the DSM (DSM-5) further refined the diagnostic criteria, categorizing ADHD into distinct subtypes, including predominantly inattentive, predominantly hyperactive–impulsive, and combined presentations. It also emphasized the disorder’s long-term impact on learning, social interactions, and emotional regulation (Austerman, 2015). The 2022 revision, DSM-5-TR, introduced key modifications, such as extending the age threshold for symptom onset, increasing attention to adult ADHD, improving cultural sensitivity, refining symptom descriptions, and highlighting the comorbid relationships between ADHD and other psychiatric disorders (Koutsoklenis and Honkasilta, 2022; Francés et al., 2023). As research progresses, our understanding of ADHD continues to deepen. This includes its symptoms, subtypes, and comorbidities. The integration of AI technologies in data analysis is becoming increasingly evidence-based. Consequently, the reliability of AI-driven screening and diagnostic models is improving (Finley et al., 2024).

The most common AI techniques used in ADHD research were machine learning, deep learning, and CNNs. Machine learning allows computers to learn from data and make predictions, while deep learning, a subset of machine learning, automatically extracts complex data features through multi-layer neural networks. CNNs are a type of deep learning architecture specifically designed for image and video analysis (Adnan and Umer, 2022; Derry et al., 2023; Li et al., 2025). By integrating hybrid AI techniques, researchers can rapidly and objectively analyze EEG, MRI, clinical symptoms, and other indicators in ADHD patients. This approach facilitates the development of screening and diagnostic models. These models have the potential to enhance the objectivity of ADHD screening and diagnosis (Amado-Caballero et al., 2023; Lee et al., 2023; Berrezueta-Guzman et al., 2024). Goh et al. (2023) employed machine learning techniques to analyze the clinical symptoms of 399 children (including both ADHD patients and non-patients) to predict future ADHD diagnosis and associated impairments. The study identified eight key symptoms (such as difficulty maintaining attention, failure to complete tasks, and distractibility) as the most significant predictors of impairment 5 years later. Notably, the AI algorithm using a simplified symptom list outperformed the algorithm that included all 18 symptoms, with an accuracy rate of 81–93%, effectively predicting both concurrent and future ADHD diagnosis (Goh et al., 2023). This study addressed the limitations of current screening tools, which have insufficient sensitivity and specificity, enhancing clinicians’ ability to identify at-risk populations for related outcomes. However, the study’s main limitations include sample biases related to race, age, and IQ, the exclusion of gender differences, and the need for further validation across diverse samples to improve the clinical applicability of the algorithm. In conclusion, while AI technologies can improve ADHD diagnosis accuracy, models still lack consistency and comprehensive validation. Future research should focus on large-sample clinical validation and model optimization to enhance clinical utility.

### 4.3 Trends of AI in ADHD

Based on the changes in research hotspots in different periods, the changing trends of research hotspots are analyzed to provide researchers with directions for future research. The applications of AI in ADHD have gradually increased. Emerging keywords such as “diagnosis,” “network,” “attention deficit hyperactivity disorder” and “artificial intelligence” and keywords cluster analysis reflect the latest research hotspots. Research trends have shifted toward the application of artificial intelligence techniques, particularly neural networks. These methods aim to identify objective diagnostic and classification indicators for ADHD. They assist in diagnosing and categorizing ADHD, including differentiating it from other neurodevelopmental and psychiatric disorders. Additionally, they contribute to the classification of ADHD subtypes. Researchers have utilized AI technologies to create diagnostic and classification models from MRI, EEG, and other data. These models enhance the accuracy of ADHD diagnosis and identification. They also demonstrate AI’s ability to refine diagnostic processes and improve our understanding of ADHD’s neurobiological underpinnings. High-impact articles have shown that various AI models demonstrate high accuracy in ADHD diagnosis and classification, with recent models achieving accuracy rates above 90%, particularly those based on CNN technology. For instance, the CNN model based on EEG data achieved an accuracy of 98.48% in classifying ADHD. In comparison, the fully connected neural network based on fMRI data reached accuracy rates of 90% for ADHD diagnosis and 95% for ADHD subtype classification. However, the majority of these studies have been validated using the ADHD-200 database, lacking large-scale clinical validation, and the models are not standardized. Therefore, the development of network-based AI technologies for diagnosing and classifying ADHD using multimodal data, along with further clinical validation to ensure their robustness and practical applicability, will be the current and future research trend.

### 4.4 Limitations of AI in ADHD

Several limitations of this study need to be acknowledged. Firstly, the exclusive use of the WoSCC database, while ensuring high-quality articles, might have omitted relevant studies not listed therein. Secondly, by focusing on articles in mainstream languages, we potentially excluded valuable research in other languages. Thirdly, the publication cycle of research may have excluded some recent studies or those not yet published. Additionally, there are several limitations in AI research and clinical applications in the field of ADHD. Firstly, AI models lack uniformity, with differences in algorithms and data processing methods, leading to a lack of standardization, which presents significant challenges for clinical implementation. The use of cross-validation methods has led to inflated performance estimates compared to those obtained from a held-out test set, which compromises the generalizability of the models. Furthermore, the heterogeneity of models makes it difficult to compare results across different studies and hinders the development of reliable and reproducible tools. Secondly, most studies are single-center studies, relying on specific datasets (e.g., ADHD-200) for validation, which may limit their generalizability to diverse clinical populations. Lastly, the process of translating AI models from research into actual clinical practice also faces challenges, particularly in terms of data quality, clinician training, and patient diversity. Despite the potential of AI in ADHD diagnosis, there remain substantial difficulties in clinical adoption, requiring further standardization and validation. Furthermore, the focus predominantly lies on screening and diagnosis, with less attention on the underlying mechanisms and intervention treatments.

To address these issues, future research should aim for broader inclusivity by incorporating multiple databases and languages. Collaborative efforts across countries, regions, institutions, and disciplines should be strengthened to facilitate large-scale, multi-ethnic studies, thereby increasing the reliability of research outcomes. Additionally, AI models need to be standardized and clinically validated, with the integration of multimodal data such as neuroimaging, genetics, and behavioral data to further improve the diagnosis accuracy. Additionally, ethical concerns, including data privacy, security, and potential biases in AI models, must be addressed to ensure the reliable application of AI technologies in clinical settings. Furthermore, expanding the research scope to include studies on mechanisms and intervention treatments will enrich the range of AI applications in ADHD.

## 5 Conclusion

This study systematically analyzed the current status of AI applications in ADHD research, identifying crucial contributors and their influence through comprehensive analysis. The analysis delineates significant partnership networks across countries, regions, institutions, and journals, encouraging researchers to engage more with influential entities to elevate research standards in this field. The examination of keywords and high-impact articles reveals current hotspots and frontiers in AI applications for ADHD, offering insights into potential future research trends. This study provides an in-depth insight into the collaborative dynamics and crucial research findings within the field. By identifying important contributors and achievements, it highlights areas of focus for researchers and outlines directions for future collaborations, fostering cooperation to accelerate the applications of AI in ADHD research.

In recent years, the applications of AI in ADHD research have emerged and continue to rise. Notable contributions have come from institutions such as the United States, the State University of New York System, and the journal *Frontiers in Psychiatry*. The focus remains on diagnosing and classifying ADHD through AI technologies that analyze MRI, EEG, ECG, and behavioral data, achieving notable accuracy. Keywords such as “diagnosis,” “network,” “attention deficit hyperactivity disorder” and “artificial intelligence” indicate current research hotspots, with a growing emphasis on network-based AI technologies for diagnostic enhancement. High-impact articles align with these trends, advocating for AI’s potential in ADHD diagnosis and classification through objective measures. Based on the above data and analysis, this study identifies the growing significance of data-driven models in diagnostic accuracy and therapeutic interventions. It also reveals the evolution of AI technologies from exploratory research to the development of diagnostic and classification tools. Additionally, it explores the current prospects of using MRI, EEG, and other data, combined with deep learning, machine learning, and CNNs technologies. These methods are applied to ADHD diagnosis, classification research, and clinical applications.

However, the field faces challenges, including model uniformity and clinical validation. Given that research combining ADHD and AI is still in its early stages, the current studies are not yet comprehensive or in-depth. Future research should address these challenges through larger-scale, multi-center studies, the integration of multimodal data, and fostering interdisciplinary collaboration. These efforts will help fully realize the potential of AI in ADHD diagnosis and treatment. Additionally, future studies must overcome technical challenges, conduct clinical validation using large datasets, and ensure AI tools are adaptable to real-world scenarios. This will enhance the accuracy of ADHD clinical screening and diagnosis, contributing to early intervention and improved prognosis. While the diagnosis of ADHD is essential for determining appropriate interventions, AI technologies are more likely to be effective in the initial screening process and the final diagnosis must still be validated by qualified clinical professionals. As research into the pathophysiological mechanisms of ADHD advances, objective diagnostic markers may be discovered. AI technologies could play a crucial role in enhancing the accuracy of ADHD diagnosis in the future.

## Data Availability

The original contributions presented in the study are included in the article/supplementary material, further inquiries can be directed to the corresponding authors.
